# The Primary Nursing Care Model and Inpatients’ Nursing-Sensitive Outcomes: A Systematic Review and Narrative Synthesis of Quantitative Studies

**DOI:** 10.3390/ijerph20032391

**Published:** 2023-01-29

**Authors:** Isabel Gonçalves, Diana Arvelos Mendes, Sílvia Caldeira, Élvio Jesus, Elisabete Nunes

**Affiliations:** 1 Universidade Católica Portuguesa, Institute of Health Sciences, Center for Interdisciplinary Research in Health, Palma de Cima, 1649-023 Lisbon, Portugal; 2 Hospital da Luz Lisboa, Avenida Lusíada, 100, 1500-650 Lisbon, Portugal; 3 Instituto Politécnico de Setúbal, Escola Superior de Saúde, NURSE’IN-UIESI, Estefanilha, 2910-761 Setúbal, Portugal; 4 Escola Superior de Enfermagem de Lisboa, Nursing Research, Innovation and Development Centre of Lisbon, 1600-190 Lisbon, Portugal

**Keywords:** inpatients, primary nursing, nursing-sensitive outcomes, patients’ experience, patients’ satisfaction

## Abstract

Background: The delivery of quality, safe, and patient-centered care is foundational for professional practice. The primary nursing model allows nurses to have excellent knowledge about patients and families and to plan and coordinate care from admission to discharge, with better management of health situations. Nurses play a crucial role in improving patients’ outcomes, namely those sensitive to nursing care. The knowledge of the relationship between the primary nursing model and the nursing-sensitive outcomes provides new scientific evidence that strengthens the relevance of this nursing care organization model in the inpatients’ health outcomes. This systematic review describes the relationship between nurse-sensitive inpatients’ outcomes and the primary nursing care model. Methods: A systematic review was conducted with a narrative synthesis, and the following databases were searched: MEDLINE, CINAHL, Web of Science, Nursing & Allied Health Collection, SciELO Collections, and Cochrane. Results: A total of 22 full texts were assessed, of which five were included in the study according to the selection criteria. The analysis results indicated that the primary nursing care model was related to nursing-sensitive patient safety outcomes. Patients’ experience was also considered a nursing-sensitive outcome, namely in the satisfaction with nursing care. Conclusion: The negative outcomes are clearly related to the primary nursing care model. There is scarce research that relates primary nursing to positive outcomes, such as patients’ functional status and self-care abilities, and more studies are needed.

## 1. Introduction

The way nurses are organized and how nursing care is delivered are critical factors for quality and patients’ outcomes in hospital stays [1]. Patients’ satisfaction with care is affected by missed nursing care and the nurses’ work environment [2]. A care model can be defined as a set of frameworks, policies, and procedures that guide nursing care [3]. A professional practice model is a basis for quality, safe and patient-centered care, for nurses’ job satisfaction and provides a theoretical background that enables nurses to explain and share their practice [4,5]. An analysis of 38 professional practice models revealed that they were based on a clearly defined conception of nursing, relation-based care, a theoretical context, and the most incorporated core organizational values [4]. Additionally, all models found six components: leadership; nurse independence and collaboration; practice environment; research/innovation; nurse development and rewards, and patients’ outcomes [4]. According to such findings, reflection on the organization of nursing care, particularly in inpatient settings, and how these can affect patient outcomes, is of great importance to care quality and effectiveness.

Looking at models of care delivery, they can be classified as (a) functional, focusing on the execution of tasks; (b) team care, where care is provided by a team guided by a leader and including professionals with different levels of competence; (c) individualized care, where each nurse is assigned to the complete care of a set of patients in each shift; and (d) primary nursing, where the responsibility of a nurse for a group of patients occurs from admission to discharge [3,6]. The term primary nursing has been seen as a generic and philosophic way of providing care. Still, it needs to be applied to explain how nurses deliver care in each specific organizational context [7]. The primary nursing model allows nurses to plan and coordinate patients’ care over time based on trust relationships; this care organization is considered essential to avoid fragmentation, improve nursing documentation and achieve person-centered quality nursing care [8,9]. As part of primary nursing, a nurse’s role is to assess health care needs, and plan, structure, and evaluate that care while the patient is at the unit; this nurse may implement the care plan or delegate it to other team members [8]. 

Within hospital environments increasingly characterized by patients’ complexity, shorter stays, high readmission rates, heavy workloads, skill mixes, and suboptimal staff, nurses’ managers may perceive primary nursing as a supportive structure for care related to patient-centered care philosophy [6,10]. Patient-centered care is associated with satisfaction; making care more adapted and focused on the patients’ needs can contribute to better outcomes [11,12].

Nursing interventions related to patients’ education seem to reduce readmissions, the causes of which are related to deficiencies in knowledge about the disease, health status, treatments, or difficulty in self-care [13]. A model such as primary nursing, which foresees the existence of a reference nurse, can be a facilitator for patients’ education. Patients seemed to be more satisfied when they knew there was a nurse responsible for their care and who was available to provide information [1]. 

Investment in nursing resources and quality practice environments have long been known as critical factors in achieving better patients’ outcomes and ensuring the quality of care [14,15]. The nurses’ performance-sensitive indicators are the result of the interaction among the available resources, the environment, the interventions, and patients’ outcomes [16]. Five nursing-sensitive outcome indicators were identified, (1) patients’ well-being, which includes interventions regarding the satisfaction of daily living needs and symptoms management; (2) safety and risk factors, which can include falls, medication errors, pressure ulcers or urinary tract infections; (3) empowerment, which reflects changes in patients’ behaviors related to nursing interventions; (4) functional status, including physical, psychosocial and cognitive status, resulting from nursing interventions; and (5) satisfaction with the care experience, which reflects the link between patients’ expectations and the perception of the actual outcomes obtained with the nursing care provided [16,17].

A systematic review that analyzed studies conducted between January 1990 and March 2013 in surgical, medical, orthopedic, and maternity settings on primary nursing as a method of care delivery and its association with patient outcomes concluded that this practice only had a positive impact on the care of women and their children in the maternity ward [18]. The authors stated that future research must attempt to relate the primary nursing model to nursing-sensitive patients’ outcomes, in particular medication errors, duration of treatments, the prevalence of infections in hospital stays, and health service utilization [18]. The studies found were scarce and not robust. Further studies with a more rigorous design would be helpful to develop research concerning the relationship between the primary nursing model on patients’ and nurses’ outcomes [19].

This systematic review clarified how the primary nursing care model is related to nursing-sensitive inpatients’ outcomes, namely the relation between nursing interventions and inpatients’ experiences. Currently, thinking about the quality of nursing care is imperative in any context. One of the possible ways to assess the quality of care is to use indicators obtained from patients related to the outcomes of nursing interventions. Other studies have identified patients’ satisfaction with respect as an indicator, but it is believed that it would be more relevant to focus on the broader concept of the patient experience. Thus, an objective and two questions were identified for this systematic review. In the first step, it was considered crucial to see whether any association was found between nursing-sensitive outcomes and this model of care for patients in hospital settings, and in the second step, an attempt was made to understand whether this model of nursing care may have any association with the inpatient experience. It is expected that the more we know about the effects that the organization of care may have on patients, the more we will be able to adapt care to their real needs and expectations.

Aims:

This systematic review described the relationship between nursing-sensitive inpatients’ outcomes and the primary nursing care model.

Review questions: 

(1) Is there any evidence regarding the association between nursing-sensitive outcomes and a primary nursing care model for inpatients?

(2) Is the primary nursing care model described as being associated with changes in the inpatients’ experience?

## 2. Materials and Methods

The guidelines of Preferred Reporting Items for Systematic Reviews and Meta-Analyses (PRISMA) were used to report on this review [20]. 

### 2.1. Search Strategy

The search approach was to find the available texts and was preceded by an exploratory search to find the most accurate terms (indexed and free) aiming for a sensitive main search according to the review goals. CINAHL and MEDLINE were the databases searched through EBSCOhost in the first phase, and all titles, abstracts, keywords, and terms considered relevant for describing the articles that were identified. Afterward, a full search was conducted in the databases using Medical Subject Headings (MeSH) and other vocabulary structured according to the databases, as well as free terms. The full search was performed in June 2021. The combined search terms were: “chronic disease” or “chronic conditions,” or “inpatients,” or “acute care” or “wards,” and “primary nursing” or “primary nursing model” and “models of nursing care” or “nursing care delivery systems” and “patients’ outcomes” or “nursing-sensitive outcomes” or “patients’ satisfaction” or “patients’ experience”. The search was updated in October 2022. The complete search strategies are provided in Appendix A. The references of the selected articles were analyzed. The complete search was carried out in the following databases: Nursing & Allied Health Collection, Web of Science Core Collection, and via the EBSCOhost platform, and the databases CINAHL Plus with Full Text and MEDLINE with Full Text, SciELO Collections, Cochrane Central Register of Controlled Trials, RCAAP (Open Access Scientific Repository of Portugal) were also searched using the following terms: inpatients, primary nursing, and patients’ outcomes. 

### 2.2. Selection Process

This review included studies (1) whose subjects were 18 years of age or older and admitted to acute care units; (2) studies concerning the use of primary nursing and inpatients’ nursing-sensitive outcomes, namely those related to nursing interventions: promoting patients’ comfort and quality of life; preventing medication-related errors, patients’ falls, pressure ulcers and urinary tract infections, patients’ empowerment, as well as functional status; and (3) studies on inpatients’ experience, namely satisfaction with nursing care.

The studies included patients from all medical and surgical specialties admitted to acute care wards. There was no limitation on the geographic location. The included studies were primary research studies using quantitative methods, namely randomized controlled trials and non-randomized controlled trials, case–control, cohort, and before-and-after studies. Secondary reviews or synthesized evidence of primary research studies, namely systematic reviews, have been excluded. Full-text studies available in the English, Spanish and Portuguese languages were considered. The search considered the date of publication from the 2000s to 2022, as the phenomenon of health outcomes and their association with nursing care began to be more systematically studied from the last decade of the 20th century [21]. Studies that solely analyzed the relation of the primary nursing care model with professionals were excluded.

### 2.3. Data Extraction

The titles and abstracts collected by the search strategy were independently analyzed by two authors (I.G. and D.A.M.) regarding the inclusion criteria to determine those eligible for full-text reading and analysis, and the results were compared. The articles selected by consensus were uploaded to Mendeley Reference Manager 2.79.0 Mendeley Ltd, read in total, and it was decided which ones were eligible for the study. The reason for exclusion is shown in the -Prisma flow diagram in the results section. The same authors collected data from the review articles using a specific tool. The information contained in the tool includes the article title and authors, year of publication, research design, settings, and participants (sample and characteristics), outcome measurement and conclusions. Disagreements at either stage were resolved through discussion until agreement. 

### 2.4. Outcomes 

The outcomes searched for in the analyzed studies were the nursing-sensitive patients’ outcomes with the primary nursing model. The outcomes were selected according to Dubois et al. [16], namely: (a) falls; (b) medication errors; (c) urinary infections; and (d) pressure ulcers. The outcomes to measure the patients’ experience are: (1) nursing interventions addressing patient self-care and safety; (2) symptom management; (3) satisfaction with nursing care; and (4) discharge planning. These results have also been agreed among several authors to be sensitive to nursing care [22,23,24].

### 2.5. Quality Assessment of Studies

According to Armijo-Olivo et al. [25], the “Effective Public Health Practice Project” (EPHPP) uses a more generic scale that allows a broader range of study designs to be assessed that includes RCTs, observational studies, cohort, case–control, or other studies. The EPHPP tool was applied to analyze the five studies’ quality. The tool incorporates six dimensions: (a) selection bias; (b) design; (c) confounders; (d) blinding; (e) data collection methods; and (f) withdrawals and drop-outs. The tool guidelines state that each component is scored as strong (1 point), moderate (2 points), or weak (3 points), and the average score for each dimension is calculated to provide the total score. According to their overall ranking, studies were allocated a quality grade of weak, moderate, or strong. The global ranking for each study was determined by considering the rankings of the six dimensions. All studies without weak scores were classified as strong. Those with one weak score were categorized as moderate. Finally, the studies with two or more weak scores were classified as weak. The minimum scores of studies to be included have not been defined previously [25,26]. Quality assessment was conducted by two independent reviewers and reviewed by three, also independently. To increase the level of reliability, the Kappa index was calculated to test the agreement between the two primary reviewers. Disagreements between the reviewers were debated until an agreement was reached. 

### 2.6. Data Synthesis and Analysis

The studies included in this systematic review were summarized in a table presenting the main characteristics of each study, including the study title, authors and year of publication, research design, settings, and participants (characterization of the hospital and sample), the instruments used in the measurement of outcomes and the main findings. The studies analyzed used different methodologies and instruments to measure the same outcomes, which made a meta-analysis unviable [27,28], so a narrative synthesis was used to present the results. 

Following the presentation of the studies’ characteristics, they were analyzed for quality regarding selection bias, study design, confounder blinding, data collection method, and withdrawals/dropouts using an assessment instrument applied to each study. The results of this analysis are presented in a table. The results of the studies were grouped to answer the purpose of the study and the research questions. Three groups were found: the first group identified the scope in which the primary nursing model of care was analyzed in each study; the second group identified the relationship between the primary nursing model of care and inpatients’ sensitive nursing outcomes; and the third group analyzed the relationship between the primary nursing model of care and inpatients’ experience. A fourth group was created concerning a set of other varied outcomes found in the studies that did not fall within the scope of this review but were considered relevant to be presented because they have an impact on professionals and may indirectly influence the analyzed outcomes.

## 3. Results

### 3.1. Study Selection

**Figure 1:**
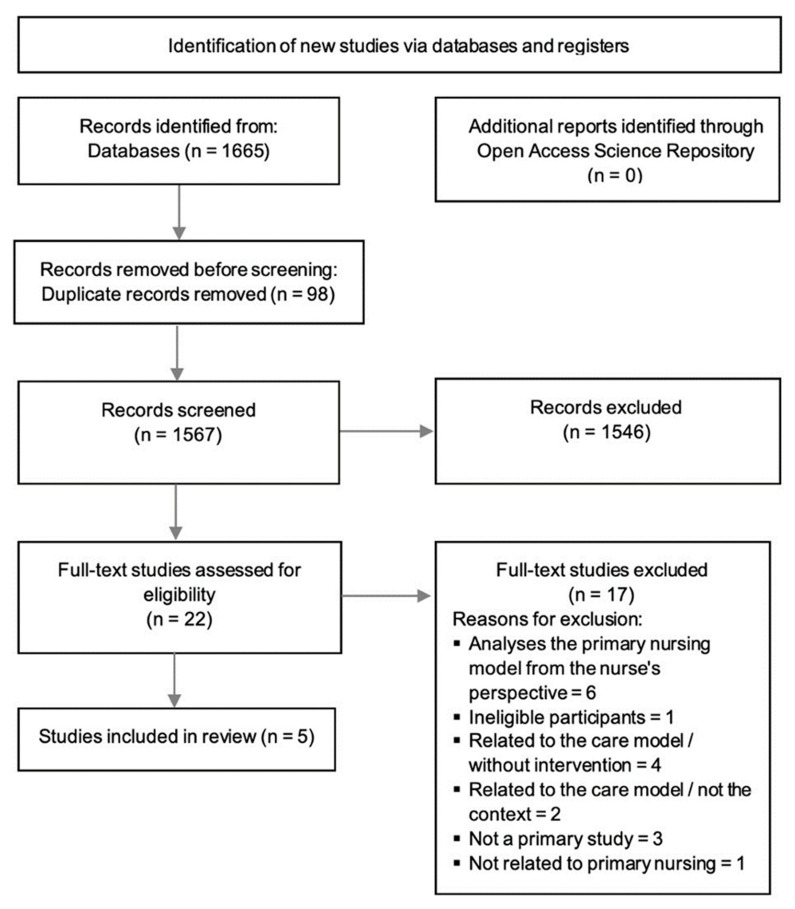
PRISMA diagram flow.

Through the search performed in the databases, 1665 papers were identified: Web of Science Core Collection = 1316, Scielo Citation Index = 46, CINAHL Plus with Full Text = 68, MEDLINE with Full Text = 201, Nursing & Allied Health Collection = 20, and Cochrane Central Register of Controlled Trials = 14. The titles and abstracts of 1567 articles were analyzed after duplicate removal. No reports were obtained through the Open Access Scientific Repository. The main reasons for excluding studies by title and abstract were that they needed to analyze the outcomes of applying the primary nursing model or focus on the hospital setting; some studies were eliminated due to inappropriate participants and the full text not available. The search findings are fully described and presented in a PRISMA flow diagram (Figure 1).

### 3.2. Study Characteristics

This systematic review included five studies [29,30,31,32,33]. All studies are recent, with three published in 2020, one in 2019, and another in 2018. Two studies were conducted in Asia, one in China and one in Israel; two are European, one from Italy and one from Switzerland; there was also a study from South America, namely Brazil. All analyses were conducted in large acute care hospitals, two of which were university hospitals. Four of the studies have samples of more than 300 participants [29,30,32,33], and one study had only 96 subjects as a sample [31]. One of the studies was a quasi-experimental study [29], and the remaining were cross-sectional studies. The average length of stay varies between 5.7 and 6 days in three studies; two studies do not mention it. The results of four studies refer to patients and nurses, and only one study reported results exclusively for patients related to primary nursing. Table 1 synthesizes the characteristics of the five selected articles.

### 3.3. Quality Assessment of Studies

Table 2 presents the authors’ final quality rating of the five studies. Two studies were classified as strong, two considered moderate, and one as weak [25,26]. Agreement on the quality of studies between authors using Kappa statistics with linear weighting, with a 95% confidence interval, was an almost perfect consensus (0.816) [34].

### 3.4. The Use of the Primary Nursing Care Model in Inpatients’ Outcomes

The effect of implementing the primary nursing care model on patients, namely nursing-sensitive outcomes, and satisfaction with care, was studied by Dal Molin et al. [29]. With the use of the model, there was a small increase in some of the nurses’ competencies, namely in the helping role, in such diagnostics, managing situations, and teaching or coaching the patients. The implementation of primary nursing ensured that (1) each patient had a designated nurse with responsibility for their nursing interventions, (2) an individual nursing care plan was developed for each patient, and (3) a discharge plan was established for patients [29]. 

The Naef et al. [30] study explored the benefits of using the primary nursing model on care coordination, patient-centered care, and care quality perceived by patients.

The authors concluded that central patients had a designated primary nurse, and admission evaluations and care planning were accomplished within 48 h; most patients had discharge planning activities documented in the records; and in about 50.0% of patients staying for seven days or more, weekly monitoring assessments and adjustments to the care plan were made by the primary nurse. Implementing the primary nursing model has reduced missing care by around 80.0 %, increasing the quality of patients’ care [31]. Likewise, Chen et al. [33] concluded that the length of the patient stay was shorter after using the primary nursing model. 

The study of Tonkikhon et al. [32], where the primary nursing model was not used, concluded that, on average, patients had allocated the same nurse less than twice and received care from seven different nurses throughout their hospitalization; 21.0% of inpatients between two and seven days were never allocated the same nurse on successive days, and 81.0% of these patients were allocated a different nurse on each work shift. The authors mentioned that dispersion in nurses’ assignments to patients interferes negatively with relational continuity, the inpatients’ experience, and the preservation of their cognitive status, especially in the elderly.

### 3.5. Primary Nursing Care Model and Inpatients’ Nursing-Sensitive Outcomes

Two studies reported the relationship between the primary nursing model and nursing-sensitive patients’ outcomes, namely: venous catheter-related infection, pressure ulcers, falls, as well as medication errors and urinary infection [29,33]. The Chen et al. [33] study, applying multilevel statistical models, found that nurses reported fewer adverse events in 2016 after implementing the primary nursing model than in 2009 before using this model, controlling for nurse-level covariates (nurses’ characteristics). Similarly, the study by Dal Molin et al. [29] also found that the incidence of adverse events decreased following the use of the primary nursing model, such as pressure ulcer, patients fall, urinary infection, infection of peripheral and central venous catheters. The study of Tonkikh et al. [32] does not relate patients’ outcomes to primary nursing, but reports that patients who had the highest care continuity, i.e., a single nurse assigned on consecutive days, was similar to the number of patients who had lower levels of disease severity, comorbidities and impaired cognitive status at discharge compared to admission baseline. The remaining two studies do not report patients’ outcomes to nursing care.

### 3.6. Primary Nursing Care Model and Inpatients’ Experience

Four of the analyzed studies reported results related to the patients’ experience with care [29,30,32,33]. Two of them addressed the satisfaction with hospital care experience [29,32]. Dal Molin et al. [29] concluded that there was an increase in patients’ satisfaction with the care provided by nurses, and the use of the primary nursing model had a medium effect on this outcome. The hospitalized patients with the highest values regarding the assignment of the same nurse for successive days showed the highest values of satisfaction with the care experience [32]. A couple of studies [30,33] refer to patients’ views on nursing care quality. According to Naef et al. [30], 96.5% of patients reported high overall nursing quality, and the attributes of responsiveness, proficiency and individuality of patient-centered nursing care scored highly (>90.0%). The coordination of care attributes was considered lower. As reported by Chen et al. [33], for patients, hospital ratings in 2016 following the adoption of the primary nursing model, with a score of 9 or 10 (scores of 8 to 10 are the best rating) increased compared to patients in 2009, controlling for patient-level covariates (length of hospital stay). Two studies analyzed the care quality through the nurses’ point of view [31,33]. Four months following the application of primary nursing model, according to the nurses there was an increase above 40.0% in the following activities, (1), ambulate thrice a day or as prescribed, (2) mobilization of patients every two hours, (3) preparation of meals for autonomous patients, (4) response to light call is initiated within five minutes, (5) attending interdisciplinary care conferences whenever they take place, and (6) sitting the patient out of bed [31]. According to Chen et al. [33], the length of patient stay in 2016, following the adoption of primary nursing model, was shorter than in 2009 before the use of this model. The rating of patients’ care quality “excellent” obtained from the nurses’ questionnaire increased 1.71 times in 2016 compared to 2009, controlling for nurse-level covariates (nurses’ characteristics). 

### 3.7. Other Outcomes

The analysis revealed that three studies reported the results of using the primary nursing model among nurses. The nurses’ level of leadership increased, the team’s environment improved, as well as the satisfaction with the performance of teamwork [29,31]. The nurses’ demographic and professional characteristics did not influence the adherence to the primary nursing model or the perception of the model’s benefits [30].

## 4. Discussion

The current paper systematically reviewed five studies to synthesize their findings regarding using the primary nursing model and its association with patients’ outcomes. Few studies on this topic were found in the literature, although the primary nursing model has been regarded as the preferred model for delivering care [18]. The studies were recent, and observational studies were the most common. Therefore, no significant and comprehensive conclusions could be reached about the influence of the nursing care organization model on patients’ outcomes. However, the analyzed studies allowed identifying some advantages of using primary nursing, namely in the continuity of care, the relationship established with patients, their relatives, to encourage self-care and reduce missing care. Adverse outcomes were associated with missing care, and these are partly a consequence of the organization of nursing care; on the other hand, patients’ engagement in their own self-care process is more effective and safer when they are encouraged by nurses with whom they build a relationship. As main results, we can conclude that there seems to be a relationship between primary nursing and adverse events between primary nursing and patients’ experience in the dimension of satisfaction with nursing care. Adverse outcomes were associated with missing care, partially a consequence of the organization of nursing care. With the primary nursing model, some advantages were identified, namely in the continuity of care, the relationship established between nurses, patients, and relatives, the encouragement to self-care, and the reduction of missing care. Patients’ involvement in their self-care process is more effective and safer when they are encouraged by nurses with whom they build a relationship [35,36,37,38]. However, the conclusions were not significant and comprehensive enough about the influence of the nursing care organization model on patient outcomes.

Two of the analyzed studies [29,33] centered on the relationship between the primary nursing care model and nursing-sensitive patients’ outcomes. The implementation of the primary nursing care model seems to relate to patients’ outcomes, namely the reduction of venous catheter-related infections, pressure ulcers falls, medication errors and urinary infections. These indicators are nursing performance-sensitive; they evaluate modifications in patients’ status because of interaction between the efficient management of nursing resources and their becoming quality services [16,39]. The analyzed papers revealed that the indicators are mostly associated with negative occurrences, such as adverse events or complications. No positive indicators related to nursing care were found, namely the patients’ involvement with health care, their functional status, or self-care skills [16]. The study of Tonkikh et al. [32] does not associate patients’ outcomes with the primary nursing model but relates the assignment of the same nurse to patients on consecutive days with lower disease severity and comorbidities as well as better cognitive status at discharge. This is consistent with recent research suggesting that when patients are encouraged, coached, and supported by nurses, they are more active and involved in their self-care during hospitalization [13]. Two studies did not analyze patients’ outcomes and their association with the primary nursing model. We could conclude from the studies’ analysis that the primary nursing care model seems to be related to nursing-sensitive patients’ outcomes, particularly to undesirable events such as adverse events.

Patients experience is one indicator of the quality of care provided throughout hospitalization; nursing care has a significant weight in this equation, and perhaps that is why four of the analyzed studies present results on this topic [29,30,32,33]. These studies indicate that the primary nursing model improves satisfaction with nursing care and, consequently, global satisfaction with a hospital stay. Satisfaction is also associated with the patients being assigned the same nurse on consecutive days. Nurses’ responsiveness, care proficiency, and individualized and patient-centered care are also valued. Previous studies highlight the importance of a nurse with responsibility for continuity of care to the satisfaction and empowerment of patients, a nurse whom the patients can identify [18,40]. No study has examined symptom management or discharge planning from the patient’s perspective. The quality of care was analyzed in two studies, but only from the perspective of nurses [31,33], who observed that by implementing the primary nursing model, they had more time to organize and deliver individualized care and were more available to patients. As a result of these changes, patients’ satisfaction with nursing care increased, and, in turn, there was a decrease in the average length of hospital stays. Satisfaction with care is an indicator that nurses increasingly value as a measure the care quality they deliver [11,41,42]. According to the studies assessed, the primary nursing model is related to the patients’ experience, particularly in the dimension of satisfaction with nursing care. They value the existence of a nurse assigned the responsibility for continuity of care.

Although this study did not aim to analyze nurses’ views on primary nursing, it was observed that three of the studies addressed it [29,30,31]. Using the primary nursing model has benefits perceived by nurses, particularly in terms of leadership, work environment, and satisfaction with teamwork. The more productive practice environments are those in which nurses can achieve greater autonomy in delivering care through monitoring some health conditions or in health education actions, which will result in positive patients’ outcomes [43,44].

This review has identified some evidence that can contribute to improving nursing care organization with a positive impact on nursing-sensitive patients’ outcomes. Political decision-makers and nurse administrators’ support for applying the primary nursing model as an organization of nursing care can be a strategy to improve professionals’ performance, and patients’ satisfaction, achieve good indicators of health quality and safety, as well as reduce patients’ average length of stay in hospitals. However, more research is needed to allow more robust and widespread results to be produced.

The limitations of this systematic review ought to be considered when reading the results. The heterogeneity of the studies included may also compromise the conclusions, viewing location and cultural dimensions of healthcare. Although they all addressed the primary nursing model, it was studied from different perspectives. Regardless of the sensitive search strategy, a small number of studies were found. In many searches, there were articles on primary care nursing instead of articles on the primary nursing model.

## 5. Conclusions

This systematic review has synthesized evidence from recent studies on the primary nursing model, its relationship to nursing-sensitive inpatients’ outcomes, and their experience with care. It was found, however, that in the studies under analysis, the indicators presented were mainly associated with negative occurrences, such as adverse events or complications. There is scarce research that relates primary nursing with positive indicators such as the patients’ functional status and self-care abilities, where the nurses’ distinctive contribution, either through health education interventions or care coordination, can be analyzed. Further research is warranted regarding the influence of the primary nursing model on these outcomes.

## Figures and Tables

**Table 1:** Characteristics of included studies.

Title/Author(s)/Publication Year	Research Design	Settings and Participants (Sample and Characteristics)	Outcome Measurement	Main Findings
“The impact of Primary Nursing care pattern: results from a before–after study.” (Dal Molin et al., 2018) [29]	Quasi-experimental (before–after study).	400-bed community hospital, Italy. t0 (before PN) = from May to November 2013); t1 (after PN) = from December 2013 to May 2014); 2857 inpatients (t0) before PN. Average age 70.4 years 3169 inpatients (t1) after PN. Average age 68 years. Adults more than 18 years old in units where PN was implemented. 369 nurses working in units where PN was implemented and that attended to the specific training sessions (82.4% female).	Patients’ outcomes: (1) Data collection tool to measure the effect of PN in patient-related outcomes: pressure ulcer; patient fall; urinary tract infection; venous catheter-related infection. (2) Newcastle Satisfaction with Nursing Scales: to measure patient satisfaction with care, the reliability of the Italian version (Cronbach’s alpha = 0.94) Nurses’ outcomes: (3) Nursing Competence Scale: 73 items and includes 7 groups of competencies (helping role, teaching-coaching, diagnostic functions, managing situations, therapeutic interventions, ensuring quality and word role); this scale exhibited good reliability (Cronbach’s alpha from 0.79 to 0.91); (4) Diagnostic Thinking Inventory: 41 items, this instrument evaluates flexibility in thinking (21 items) and the structure of memory (20 items); the overall reliability was 0.84 for internal consistency, 0.73 for flexibility and 0.75 for structure items. For the organization: (5) Empowering Leadership Questionnaire: was used to analyze head nurse leadership in 5 factors (learning by example, participative decision-making, coaching, informing and showing concern/interacting with the team); (6) Team Climate Inventory: 38 items that evaluate 4 factors (participate safety, support to innovation, vision of group and task orientation).	• The incidence of adverse events such as pressure ulcer (t0 = 136 to t1 = 126), patient falls (t0 = 67 to t1 = 59), urinary tract infections (t0 = 153 to t1 = 133) and venous catheter infection (peripheral t0 = 61 to t1 = 30, central t0 = 12 in 215 to t1 = 3 in 295) decreased after the implementation of PN. • The nurses reported an increase in their competencies such as: helping role (t0 = 18.11 and t1 = 19.85, *p* = 0.0001); diagnostic functions (t0 = 18.88 and t1 = 20.11, *p* = 0.0007); managing situations (t0 = 27.60 and t1 = 28.60, *p* = 0.0210); ensuring quality (t0 = 16.03 and t1 = 16.93, *p* = 0.0057); teaching-coaching the patient (t0 = 14.60 and t1 = 15.55, *p* = 0.0002); teaching-coaching the family (t0 = 5.60 to t1 = 5.98, *p* = 0.0015); teaching-coaching the student (t0 = 4.72 to t1 = 5.14, *p* = 0335); teaching-coaching evaluation of the education program (t0 = 8.29 and t1 = 8.90, *p* = 0.0004). • With the application of Diagnostic Thinking Inventory, the results were an increase of thinking flexibility (from 92.39 to 96.34, *p* < 0.00001) and structure of memory (from 86.49 to 92.16, *p* < 0.00001).
“Adaption, benefit and quality of care associated with primary nursing in an acute inpatient setting: A cross-sectional descriptive study” (Naef et al., 2019) [30]	Descriptive cross-sectional study.	900-bed University hospital In Switzerland, 2017 study. Acute care inpatients. N = 369 inpatients Median age = 59 years Female = 52.0% Median hospital stay = 6 days N = 381 nurses N = 360 registered nurses N = 13 practical nurses (Swiss certification) N = 8 nurse assistants Female = 48.2% Full-time work = 246 Registered nurses = 60.8% Bachelor’s degree/Master of Advanced Studies = 33.6% Postgraduate degree = 5.6% Worked as primary nurses = 87.8% registered nurses	(1) Primary Nursing Scale (PNS)—a nine-item questionnaire (nurses’ perception of the extent to which primary nursing was adopted and was beneficial in ensuring coordinated, person-centered care in an acute care setting). A Likert scale 1–6, was used and high scores indicated high level of primary nursing adoption and benefit; (2) A structured extraction sheet to uptake primary nursing in the patient care process, as evidenced in the patient record, with four indicators dichotomized (yes, no): (a) a primary nurse had been assigned to the patient; (b) nursing admission assessment and care planning had been completed within 48 h by the primary nurse; (c) follow-up assessment and care planning had been conducted at least once a week by the primary nurse; (d) discharge planning had been initiated by the primary nurse; (3) The Oncology Patients’ Perceptions of Quality of Nursing Care Scale (OPPQNCS). The 18-item OPPQNCS measures patients’ perceptions of the quality of nursing care in four types of person-centered nursing interventions: responsiveness; individualization, coordination, and proficiency (Likert scale 1–6 was used, high scores indicate higher quality of person-centered care).	• 96.5% of patients reported high overall quality of nursing care (median score of 5.4), the attributes of patient centered nursing interventions responsiveness, proficiency and individuality of care scored high (>90.0%). The attribute coordination of care was perceived to be lower (median = 4.7); • 72.1% of patients had a primary nurse assigned to them; • 81.1% of cases had admission assessments and care planning completed within 48 h; • In 26.1% of cases, the assigned primary nurse fully completed the admission assessment and care planning; • 86.5% of patients had discharge planning activities documented in the records; • In 50.5% of patients with a stay of 7 days or longer weekly follow-up assessments and care planning by a designated primary nurse occurred; • 63.3% of nurses agreed or strongly agreed that primary nursing was adopted on their unit; • 80.5% of nurses agreed or strongly agreed that primary nursing is beneficial for the delivery of person centered care; • Nurses’ demographic and professional characteristics did not influence nurses’ perceptions of the extent of adoption or benefit of primary nursing; • There was a statistically significant positive correlation between adoption and benefits scores (Spearman’s correlation: rs 0.449, *p* < 0.000).
“Relationship between the implementation of primary nursing model and the reduction of missed nursing care.” (Moura et al., 2020) [31]	Predictive correlational study.	University Hospital in the northeast region of Brazil, 201 beds. Four inpatient units. 4 and 7 months following the implementation of primary nursing model Final sample 96 participants (37 nurses and 57 nursing technicians). Average age of 34.9 years and 88.5% female. 40.6% had nursing specialties. 96.9% worked full time in the unit. 58.3% worked 6-h shifts, 36 h per week. Average years of experience = 8.3. Average years of experience in this unit = 1.8. 94.8% did not intend to leave their role at the unit.	MISSCARE instrument: the 56-item consisted in two parts: (A) missed care with 28-items and (B) reasons, also with 28-items. The responses had a 5-point Likert-type scale. The B part was distributed in 5 dimensions: communication (10-items), material resources (4-items), labor resources (8-items), ethical dimension (3-itemsn) and management/leadership style (3-items).	• Missed nursing care was reduced 78.5% with primary nursing. • In the fourth month of implementation of MISSCARE, 6 items obtained high levels (>40%), such as: Item 1—Ambulation three times per day or as ordered, Item 2—Turning patient every 2-h, Item 4—Setting up meals for patients who feed themselves, Item 19—Response to call light is initiated within 5 min., Item 22—Attend interdisciplinary care conferences whenever held and Item 27—Sitting the patient off the bed. Main reasons for missed care found in this study: labor resources (89.6%) and communication dimension (77.1%).
“The nurse outcomes and patient outcomes following the High-Quality Care Project” (Chen et al., 2020) [33] “Association between continuity of nursing care and older adults’ hospitalization outcomes: A retrospective observational study” (Tonkikh et al., 2020) [32]	Analytical cross-sectional study. Two studies on the High-Quality Care Project that implemented primary nursing: before (2009) and after (2016).	40 units of 10 tertiary hospitals in China 2006 study: 354 patients Male = 56.8% Mean age = 54.4 years Median hospital stay = 6 days 580 nurses Female = 98.8% Non-permanent employment contracts = 65.3% Advanced diploma = 56.2% Baccalaureate = 38.4% Master or above = 0.7% 2016 study: 550 patients Male = 59.1% Mean age = 56 years Median hospital stay = 5 days 796 nurses Female = 94.3% Non-permanent employment contracts = 73.8% Advanced diploma = 25.4% Baccalaureate = 72.0% Master or above = 2.4%	Nurse outcomes: (1) Nurse Work Index—Practice: Environment Scale (NWI-PES)—31 items divided into five subscales. A 4-point Likert scale where a higher score means a better nurse work environment; (2) Maslach Burnout Inventory (MBI)—22 items divided into three subscales. A 6-point Likert scale, a score on the Emotional Exhaustion subscale ≥27 indicate a high level of burnout; (3) An overall job satisfaction item and eight individual items of different aspects of job satisfaction (each item was scored from 0 “Very dissatisfied” to 3 “Very satisfied”); intention to leave job (measured with a dichotomous item (yes, no)). Patient outcomes: (4) Quality of patient care through an item in the nurses’ questionnaire (rating from 0 “poor” to 3 “excellent”) and patient safety by asking nurses to estimate the frequency of nursing-sensitive events, namely medication administration errors, pressure ulcers, falls, urinary tract infections and venous catheter-related infections (the rating ranged from 0 to 30, the higher the rating the higher the frequency of adverse events); (5) Consumer Assessment of Healthcare Providers and Systems (CAHPS). Scores 0 to 10 given by the patient. The scores 8 and 10 are the best rating.	• The length of patient stay in 2016 was shorter than in 2009; • The rating quality of patient care ‘excellent’ had increased by 1.718 times in 2016 compared with 2009, OR= 1.718 (*p* = 0.005), controlling the covariates on the nurse level (nurses’ characteristics); • Nurses in 2016 reported fewer patient adverse events then 2009 β = −0.894 (*p* < 0.001), controlling the covariates on the nurse level (nurses’ characteristics); • For patients, the rating of hospital in 2016 with a score of “9” or “10”, had increased compared with patients in 2009. OR= 1.705 (*p* = 0.01), controlling the covariate on the patient level (length of hospital stay).
	Retrospective observational study.	Two tertiary hospitals (internal medicine units) in Israel between 2009 and 2011 609 patients aged ≥ 70 years Mean age = 79 years Median hospital stay = 5.7 days 37.8% experienced cognitive decline 22.3% experienced physical functioning decline between admission and discharge 40.6%) reported high satisfaction with the hospital care experience.	(1) 10-item Short Portable Mental Status Questionnaire (SPMSQ), score 0 to 10 with higher scores indicating better cognitive status; (2) 11-item modified Barthel index, total score ranges from 0 to 100 (decline in physical functioning was defined as at least a 2-point decline on the modified Barthel index from the at-admission to at-discharge assessments); (3) Modified version of the Perceived Hospital Environment Quality Index at discharge with 12 of the original items, 5-point Likert-type scale, score 1 “totally disagree” to 5 “totally agree” (average score of 4 or above was considered high satisfaction); (4) Continuity of Care Index (CoC) and Sequential Continuity Index (SECON) to measure continuity in the assignment of nurses to patients: the continuity score was dichotomized into higher and lower than 75.0% and the cut-off was 25.0% (two-thirds of the sample fall within the 25.0% of the highest feasible continuity score).	• On average, patients met the same nurse 1.5 times during hospitalization. • On average, seven different nurses were assigned to care for each patient during the hospitalization; • Mean for continuity score was low for both CoC (0.09) and SECON (0.24); • 21.0% of patients hospitalized for 2 to 7 days were not assigned to the same nurse on any of the consecutive days (SECON = 0); • 81.6% of those patients were assigned to a new nurse each morning and evening shift (CoC = 0); • 31.5% achieved 25.0% of the highest feasible in-hospital CoC; • 41.2% achieved 25.0% of the highest feasible SECON; • Patients achieving 25.0% of the highest feasible in-hospital continuity were similar to patients with lower continuity levels in terms of illness severity, comorbidities, baseline cognitive and physical status and length of stay; • 25.0% of the maximum CoC was associated with lower odds of cognitive decline (OR = 0.64, 95% CI) and higher odds of high satisfaction with the hospital care experience (OR = 1.52, 95% CI); • 25.0% of the maximum SECON was associated only with higher odds of high satisfaction with the hospital care experience (OR = 1.43, 95%CI); • No significant associations were found between the CoC and SECON and decline in physical functioning.

**Table 2:** Studies quality assessment EPHPP.

	A (Selection Bias)	B (Study Design)	C (Confounder)	D (Blinding)	E (Data Collection Method)	F (Withdrawals/Dropouts)	Global Rating
(Dal Molin et al., 2018) [29]	2	1	3	2	1	NA	2
(Moura et al., 2020) [31]	3	3	1	1	1	NA	3
(Naef, Ernst, and Petry, 2019) [30]	2	3	1	1	1	NA	2
(Chen, 2020) [33]	1	2	1	1	1	NA	1
(Tonkikh, Zisberg, and Shadmi, 2020) [32]	2	2	1	1	1	NA	1
1—Strong; 2—Moderate; 3—Weak; NA—Non-applicable.							

## Data Availability

Not applicable.

## Appendix A

**Table A1:** Search terms used.

Database-	Search Strategy
Web of Science Core Collection Date of search: June 2021 and updated in October 2022	# 1 ALL = chronic disease; # 2 ALL = chronic illness; # 3 ALL = long term conditions; # 4 ALL = chronic conditions; # 5 ALL = Inpatients; # 6 ALL = Acute care; # 7 ALL = wards; # 8 #7 OR #6 OR #5 OR #4 OR #3 OR #2 OR #1; # 9 ALL = primary nursing; # 10 ALL = primary nursing model of care; # 11 ALL = primary nursing care model; # 12 #11 OR #10 OR #9; # 13 ALL = patient outcomes; # 14 ALL = nursing sensitive outcomes; # 15 ALL = patient satisfaction; # 16 ALL = patient experience; # 17 ALL = medication errors in nursing; # 18 ALL = falls in hospitalized patients; # 19 ALL = pressure ulcers in hospital; # 20 ALL = urinary tract infections catheter-related; # 21 ALL = Patient-selfcare; # 22 ALL = Patient self management; # 23 ALL = patient safety; # 24 ALL = symptom management; # 25 ALL = discharge planning; # 26 #25 OR #24 OR #23 OR #22 OR #21 OR #20 OR #19 OR #18 OR #17 OR #16 OR #15 OR #14 OR #13; # 27 #26 AND #12 AND #8 Refined by: WEB OF SCIENCE CATEGORIES: (NURSING) AND LANGUAGES: (ENGLISH OR SPANISH OR PORTUGUESE) AND DOCUMENT TYPES: (ARTICLE). (Results = 1188)
CINAHL (EBSCO host platform) Date of search: June 2021 and updated in October 2022	S1 (MH “Chronic Disease”) S2 chronic disease or chronic illness or long term conditions or chronic conditions S3 (MH “Inpatients”) S4 inpatients or hospitalization or ‘hospitalized patients’ or acute or ward or hospital or unit S5 S1 OR S2 OR S3 OR S4 S6 “primary nursing” S7 “primary nursing care model” S8 “primary nursing model” S9 “primary nursing care” S10 “primary nursing care delivery mode” S11 S6 OR S7 OR S8 OR S9 OR S10 S12 patient outcomes or quality of care or health outcomes or patient satisfaction or patient experience S13 “nursing sensitive outcomes” S14 S12 OR S13 S15 S5 AND S11 AND S14 S16 (MH “Medication Errors”) S17 “medication errors in nursing” S18 (MH “Accidental Falls”) S19 “falls in hospitals” S20 (MH “Pressure Ulcer”) S21 “pressure ulcers in hospitals” S22 “urinary tract infections, catheter-related” S23 (MH “Urinary Tract Infections, Catheter-Related”) S24 patient self-care or self management S25 (MH “Patient Safety”) S26 patient safety or patient outcomes or quality of care or safety S27 symptom management or symptom control S28 (MH “Discharge Planning”) S29 discharge planning or discharge process S30 S16 OR S17 OR S18 OR S19 OR S20 OR S21 OR S22 OR S23 OR S24 OR S25 OR S26 OR S27 OR S28 OR S29 S31 S15 AND S30 S32 S15 AND S30 Limiters—Publication Date 2000 01 01–2022 12 31; Age Groups: All Adult; Languages: English or Spanish or Portuguese (Results = 68)
MEDLINE with Full Text (EBSCOhost platform) Date of search: June 2021 and updated in October 2022	S1 (MH “Chronic Disease”) S2 chronic disease or chronic illness or long term conditions or chronic conditions S3 (MH “Inpatients”) S4 inpatients or hospitalization or ‘hospitalized patients’ or acute or ward or hospital or unit S5 S1 OR S2 OR S3 OR S4 S6 primary nursing S7 primary nursing care model S8 primary nursing care delivery model S9 primary nursing model S10 S6 OR S7 OR S8 OR S9 S11 (MH “Patient Outcome Assessment”) S12 patient outcomes or quality of care or health outcomes or patient satisfaction or patient experience S13 “nursing sensitive outcomes” S14 (MH “Medication Errors”) S15 “medication errors in nursing” S16 (MH “Accidental Falls”) S17 “falls in hospitalized patients” S18 (MH “Pressure Ulcer”) S19 “pressure ulcers in hospitals” S20 (MH “Urinary Tract Infections”) S21 “urinary tract infections, catheter-related” S22 Patient- selfcare or self management S23 (MH “Patient Safety”) S24 patient safety S25 symptom management or symptom control S26 discharge planning or discharge process or discharge management S27 S13 OR S14 OR S15 OR S16 OR S17 OR S18 OR S19 OR S20 OR S21 OR S22 OR S23 OR S24 OR S25 OR S26 S28 S5 AND S10 AND S27 Limiters—Publication Date 2000 01 01–2022 12 31; Age Groups: All Adult; Languages: English or Spanish or Portuguese (Results = 110)
Nursing & Allied Health Collection (EBSCOhost platform) Date of search: June 2021 and updated in October 2022	S1 chronic disease or chronic illness or long term conditions or chronic conditions S2 inpatients or hospitalization or ‘hospitalized patients’ or acute or ward or hospital or unit S3 S1 OR S2 S4 primary nursing S5 primary nursing care model S6 primary nursing care delivery model S7 primary nursing model S8 S4 OR S5 OR S6 OR S7 S9 patient outcomes or quality of care or health outcomes or patient satisfaction or patient experience S10 nursing sensitive outcomes S11 medication errors in nursing S12 falls in hospitalized patients S13 pressure ulcers in hospitals S14 urinary tract infections, catheter-related S15 Patient-selfcare or self management S16 patient safety S17 symptom management or symptom control S18 discharge planning or discharge process or discharge management S19 S9 OR S10 OR S11 OR S12 OR S13 OR S14 OR S15 OR S16 OR S17 OR S18 S20 S3 AND S8 AND S19 S27 Limiters—Publication Date 2000 01 01–2022 12 31; Languages: English or Spanish or Portuguese (Results = 20)
Cochrane Central Register of Controlled Trials (EBSCOhost platform) Date of search:June 2021 and updated in October 2022	S1 chronic disease or chronic illness or long term conditions or chronic conditions S2 inpatients or hospitalization or ‘hospitalized patients’ or acute or ward or hospital or unit S3 S1 OR S2 S4 primary nursing S5 primary nursing model S6 S4 OR S5 S7 patient outcomes or quality of care or health outcomes or patient satisfaction or patient experience S8 medication errors in nursing S9 falls in hospitalized patients S10 pressure ulcers in hospitals S11 urinary tract infections, catheter-related S12 Patient- selfcare or self management S13 patient safety S14 symptom management or symptom control S15 discharge planning or discharge process S16 S7 OR S8 OR S9 OR S10 OR S11 OR S12 OR S14 OR S15 S17 S3 AND S6 AND S16 Limiters—Publication Date 2000 01 01–2022 12 31 Languages: English or Spanish or Portuguese; (Results = 14)
Scielo Citation Index (Web of Scicence) Date of search: June 2021 and updated in October 2022	# 1 TS = chronic disease; # 2 TS = chronic illness; # 3 TS = long term conditions; # 4 TS = chronic conditions; # 5 TS = Inpatients; # 6 TS = Acute care; # 7 TS = wards; # 8 #7 OR #6 OR #5 OR #4 OR #3 OR #2 OR #1; # 9 TS = primary nursing; # 10 TS = primary nursing model of care; # 11 TS = primary nursing care delivery model; # 12 #11 OR #10 OR #9; # TS = patient outcomes; # 14 TS = Nursing sensitive outcomes; # 15 TS = patient satisfaction; # 16 TS = patient experience; # 17 TS = Patient reported outcomes; # 18 TS = medication errors; # 19 TS = falls; # 20 TS = pressure ulcers; # 21 TS = urinary tract infections; # 22 TS = Patient- selfcare; # 23 TS = patient safety; # 24 TS = symptom management; # 25 TS = discharge planning; # 26 #25 OR #24 OR #23 OR #22 OR #21 OR #20 OR #19 OR #18 OR #17 OR #16 OR #15 OR #14 OR #13; # 27 #26 AND #12 AND #8; # 28 #26 AND #12 AND #8 Refined by: document types: (research article) (Results = 46)
